# A Symmetric U-Shaped Gate Tunnel FET-ISFET Hybrid Label-Free Biosensor for Highly Sensitive DNA Detection

**DOI:** 10.3390/s26041337

**Published:** 2026-02-19

**Authors:** Yourui An, Yang Li, Shupeng Chen, Shulong Wang, Zhenhao Wen, Xiaoli Yang, Hongxia Liu

**Affiliations:** 1 Key Laboratory of Wide Bandgap Semiconductor Materials, Faculty of Integrated Circuit, Ministry of Education, Xidian University, Xi’an 710071, China; 23111213718@stu.xidian.edu.cn (Y.A.); slwang@xidian.edu.cn (S.W.); wenzhenhao1204@163.com (Z.W.); hxliu@mail.xidian.edu.cn (H.L.); 2 Department of Clinical Laboratory, Xi’an Central Hospital, Xi’an 710003, China; 3 Key Laboratory of Intelligent Analysis and Decision on Complex Systems, School of Mathematics and Statistics, Chongqing University of Posts and Telecommunications, Chongqing 400065, China; yangxl@cqupt.edu.cn

**Keywords:** ISFET biosensor, tunnel field-effect transistor, biosensing, TCAD

## Abstract

Ion-Sensitive Field-Effect Transistors (ISFETs) have been extensively used to detect various biomolecules, as the intrinsic charge of these molecules can change the transistor’s current or threshold voltage. Recently, realizing ISFET biosensors with better performance has attracted much attention. This paper proposes a novel ISFET biosensor by using the advantage of Tunnel Field-Effect Transistor (TFET). The device characteristics and sensing performance are systematically investigated by Silvaco Atlas TCAD simulations. Due to the novel structural design, the proposed sensor achieves a maximum current sensitivity (*S_IDSmax_*) of 99.99% and a threshold voltage sensitivity (*S_VTH_*) of 124%. To provide optimization guidelines, this work further explored the effect of geometric dimensions and gate dielectric materials on device performance. The excellent performance of the proposed biosensor makes it a promising candidate for future low-power, high-sensitivity biodetection applications.

## 1. Introduction

Biosensors possess the remarkable ability to convert biological responses into measurable electrical signals. Consequently, they have evolved into an indispensable analytical tool within modern biomedical applications, proving particularly vital for scenarios that demand large-scale biomolecular analysis [1,2,3,4]. Generally, these devices can be categorized based on their detection mechanism into two distinct types: labeled and label-free biosensors. Labeled biosensors typically necessitate the attachment of detectable markers—such as fluorescent molecules or radioisotopes—onto either the biorecognition element or the target analyte. The presence or state of biomolecules is then inferred by monitoring these markers. However, it is undeniable that labeled biosensors face significant hurdles. The detection process is often complex and frustratingly time-consuming. Furthermore, alarmingly, certain markers (such as radioactive elements) pose serious safety hazards [5]. In contrast, label-free biosensors offer a new alternative. They eliminate the need for any external tagging of the biorecognition element or the target. Instead, they achieve detection by directly monitoring the physicochemical signal changes—such as variations in electrical signals—triggered by intrinsic biomolecular interactions (e.g., antigen–antibody binding or nucleic acid hybridization) [6]. As a result, these label-free options are inherently safer and more convenient. Today, many label-free biosensors built around the Tunnel Field-Effect Transistor (TFET) operate primarily through two distinct mechanisms: one based on dielectric modulation, and the other utilizing the structure of Ion-Sensitive Field-Effect Transistor (ISFET). However, current dielectric-modulated TFET biosensors face several critical challenges. The first limitation lies in the inherent trade-off between the nanocavity dimensions and detection sensitivity. Specifically, expanding the cavity size inevitably leads to an increase in the overall device footprint. For TFETs, such dimensional scaling-up typically results in a degradation of *I_on_*/*I_off_* ratio and an increase in the Subthreshold Swing (SS), thereby compromising the overall sensitivity. Another significant issue is the ambiguity associated with dielectric modulation. On one hand, it is difficult to distinguish between biomolecules possessing similar dielectric constants; on the other hand, it remains challenging to decouple whether the variations in electrical parameters originate from the biomolecule concentration or their intrinsic dielectric properties [7].

The ISFET was originally proposed back in 1970 for monitoring ion concentration in aqueous solutions [8,9]. Interestingly, it has garnered increasing attention in recent years due to its outstanding performance in biosensing applications [10]. A traditional ISFET is composed of a fixed source, a drain, a conduction channel, and a reference gate immersed in an electrolyte [11]. The architectural design of this device aligns closely with that of a standard MOSFET. Consequently, current ISFET development predominantly leverages the well-established MOSFET fabrication processes, leading to the widespread adoption of this specific structural framework [12,13,14]. In a notable development in 2016, Guangyu Xu and colleagues introduced a Complementary Metal-Oxide-Semiconductor (CMOS) based ISFET [15] biosensor. In this innovative design, the gate is not directly exposed to the electrolyte; instead, it employs a floating gate structure capped with an oxide layer, which serves as the direct contact interface with the fluid. Crucially, this approach allows for the customization of the gate’s geometry, enabling researchers to optimize the device structure for superior biological detection outcomes.

To achieve higher sensitivity in ISFETs, it is desirable that a minute variation in *V_GS_* yields a substantial change in the drain current (*I_DS_*), thereby enabling the capture of weak biological signals. The TFET has emerged as one of the most promising candidates for this purpose, owing to its extremely low SS (below 60 mV/dec) [16]. Admittedly, other devices with low SS characteristics currently exist. For instance, the Feedback Field-Effect Transistor (FBFET) [17] also exhibits a low SS; however, it suffers from significant short-channel effects when the device dimensions are scaled down below 40 nm. Similarly, the Negative Capacitance TFET (NCTFET) demonstrates a low SS and is compatible with existing MOSFET processes, yet it still faces challenges regarding ferroelectric material stacking and material selection [18]. In contrast, TFETs are not only compatible with current MOSFET manufacturing processes [19] but also allow for further dimensional scaling. Therefore, TFETs possess distinct advantages in terms of process integration and scalability.

Based on these advantages, we propose a symmetric U-shaped gate tunnel FET-ISFET (SU-ISFET) hybrid biosensor. This biosensor achieves a remarkable current sensitivity of up to 99.99% and a threshold voltage sensitivity of up to 124%. Section 2 discusses the device structure and simulation methodology. Section 3 provides a comprehensive analysis of the simulation results, covering sensitivity optimization, noise characteristics, and process reliability.

## 2. Device Structure and Simulation Methodology

**Figure 1:**
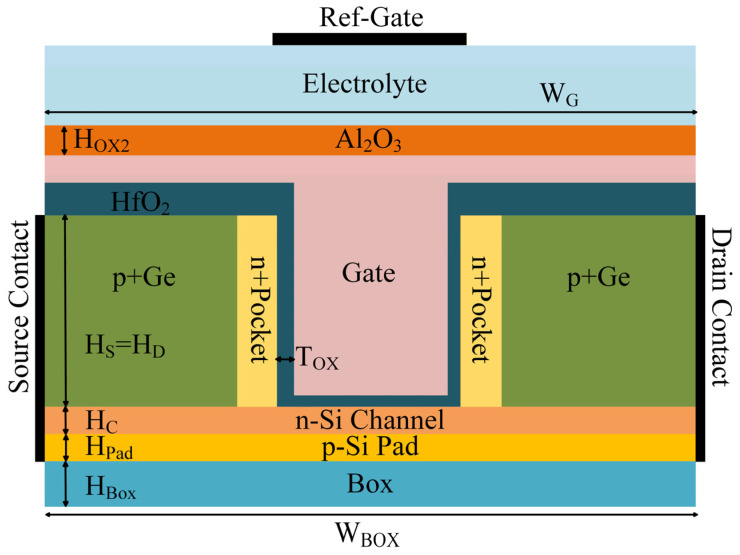
Schematic cross-sectional view of the proposed SU-ISFET.

The cross-sectional architecture of the SU-ISFET biosensor is depicted in Figure 1. In an effort to aggressively improve the Subthreshold Swing (SS), the device incorporates a high-k Hafnium Oxide (HfO_2_) gate dielectric, which adopts a distinctive “U-shaped” configuration. Furthermore, to maximize the surface area available for probe attachment, the upper segment of the gate structure has been intentionally elongated. The device features p+ Germanium (Ge) source and drain regions symmetrically positioned flanking the gate, while two n+ pocket layers are inserted to enhance the tunneling efficiency within the transistor channel. Underlying this gate structure are an n- Silicon (Si) channel and a p- Si pad layer. Strikingly similar to a conventional MOSFET, the SU-ISFET exhibits bidirectional current flow capabilities. This intrinsic characteristic renders it exceptionally well-suited for integration into large-scale ISFET biosensing arrays [12].

**Figure 2:**
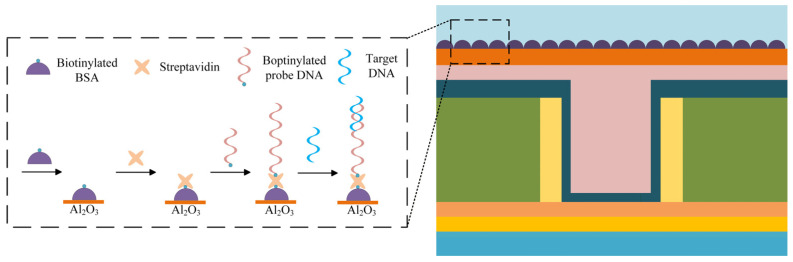
Schematic illustration depicting the surface functionalization workflow for probe immobilization on the oxide layer.

On the top of the gate lies a thin Aluminum Oxide (Al_2_O_3_) layer, specifically engineered to serve as the anchor point for DNA probes. The immobilization protocol, illustrated in Figure 2, relies on a cascade of biochemical interactions. The process initiates by immersing the sensor surface in a solution containing biotin-labeled Bovine Serum Albumin (BSA). Here, the BSA molecules robustly adhere to the oxide surface via non-covalent interactions—such as hydrogen bonding and electrostatic forces—occurring between their amino/carboxyl groups and the hydroxyl groups on the Al_2_O_3_. Once this foundation is laid, the sensor is exposed to a streptavidin solution, which binds stably to the BSA-modified interface [15]. Ultimately, when the probe DNA solution is introduced, the strands bind to the unsaturated sites on the streptavidin, thereby firmly locking the probes onto the oxide surface.

**Figure 3:**
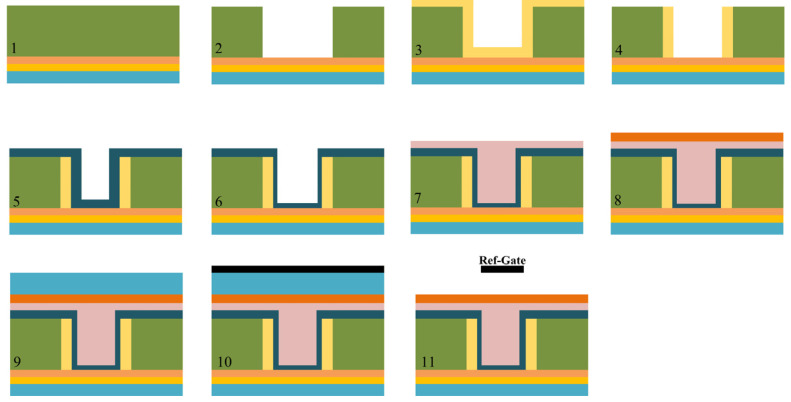
Key fabrication process flow of the proposed SU-ISFET.

Despite its unique geometry, the proposed SU-ISFET architecture maintains full material and process compatibility with standard CMOS fabrication protocols. The feasibility of realizing such structures has been substantiated by Kim et al. [20], who demonstrated the fabrication of an L-shaped TFET. Figure 3 illustrates the key steps of the proposed manufacturing flow. The fabrication sequence commences with the epitaxial growth of a silicon p-PAD layer, followed by the formation of an n-type channel region. Subsequently, a Germanium (Ge) layer is deposited to form the source and drain regions. Following the patterning of the source and drain layers via etching, a thin n+ silicon film is deposited using Chemical Vapor Deposition (CVD). An etching step is then employed to remove the excess silicon, defining the n+ silicon pocket. Thereafter, a layer of HfO_2_ is deposited to serve as both the gate dielectric and the isolation layer. To optimize device performance, the HfO_2_ layer in the gate region is thinned through the etching process. The titanium metal gate is then deposited and planarized utilizing Chemical Mechanical Polishing (CMP). Subsequently, an aluminum oxide Al_2_O_3_ layer is deposited on the gate surface via Atomic Layer Deposition (ALD) to function as the top gate oxide. Next, a SiO_2_ sacrificial layer is grown on top of the aluminum oxide, followed by the formation of the reference gate. Finally, the sacrificial layer and the excess reference gate material are removed through etching, thereby completing the fabrication of the SU-ISFET structure. It is important to note that while the reference gate appears suspended without support in the 2D cross-sectional schematic, it is mechanically anchored to the oxide layer in the actual three-dimensional device structure.

**Figure 4:**
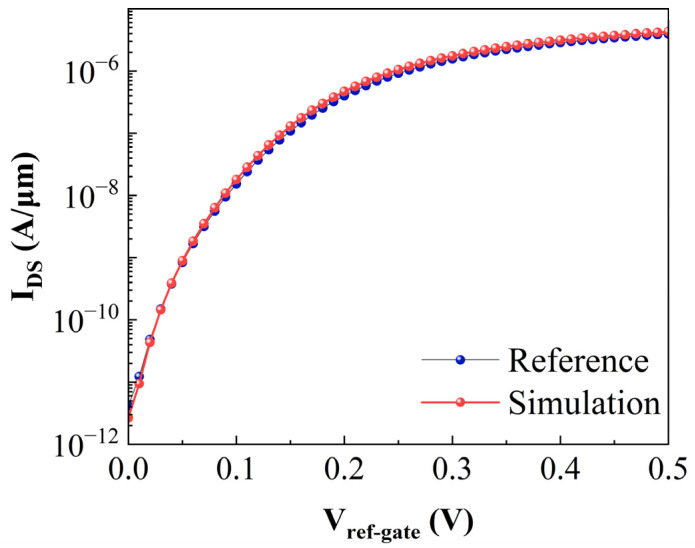
Reproduction of the SU-TFET [21] transfer characteristics using the calibrated simulation framework.

Numerical investigations were conducted using the Silvaco Atlas TCAD suite. To capture the intricate device physics with high fidelity, the simulation framework integrates a comprehensive set of models: the non-local band-to-band tunneling (BTBT) model, Fermi-Dirac statistics, the trap-assisted tunneling (TAT) mechanism, the shockley-read-hall (SRH) recombination model, band gap narrowing (BGN), and the Lombardi mobility model (CVT). In Figure 4, the transfer characteristic values extracted from ref [21] are plotted alongside our reproduced results. Excellent agreement is observed across all bias points. This close match between the reference data and our simulation results validates the accuracy of our framework, ensuring that the subsequent performance evaluations are reliable.

Modeling the electrolyte interface posed a unique challenge, which we addressed by defining a custom material. Specifically, the electrolyte is treated as a pseudo-semiconductor. We explicitly acknowledge that this approach simplifies certain physical aspects, such as specific ion adsorption and complex site-binding kinetics. Nevertheless, for the purpose of evaluating threshold voltage shifts and sensitivity trends, this modeling approach provides sufficient quantitative accuracy. Key parameters were assigned as follows: a bandgap (*E_g_*) of 1.5 eV, a dielectric constant of 80, and an electron affinity of 3.9 eV [22]. Regarding the effective density of states for the conduction (*N_C_*) and valence (*N_V_*) bands, these values are derived via Equation (1) [23]:

(1) $$N_{C}=N_{V}=\left\{\begin{array}{c} 10^{-3}\cdot N_{av}\left(c_{0}+cH_{B}\right),\;\;\;\;\;\;\;for\;pH_{B}\;\leq \;7\;\\ 10^{-3}\cdot N_{av}\left(c_{0}+10^{-14}/cH_{B}\right),\;\;\;\;for\;pH_{B}\;>\;7\;\;\;\;\;\;\;\;\;\; \end{array}\;\;\;\right.$$

Here, *N_av_* signifies Avogadro’s constant—fixed at 6.02214 × 10^23^. And *c*_0_ denotes the molar concentration of the constituent salt ions. The term *c_HB_*, defined as 10^−*pHB*^, represents the hydrogen ion concentration normalized against a 1 Molar standard solution. To mitigate the Debye screening effect and enhance detection sensitivity, the simulation environment is configured with a neutral pHB of 7, mimicking a low-ionic-strength solution. Under these specific conditions, the molar concentration of salt ions is set to 10 mM. Injecting these parameters into the equation yields the precise magnitudes for the effective density of states in both the conduction and valence bands.

The key device parameters of the SU-ISFET biosensor adopted in the simulations are summarized in Table 1.

## 3. Simulation Results and Discussion

### 3.1. Device Operating Mechanism and Basic Characteristics

The threshold voltage of ISFET can be described by Equation (2) as follows [24]:

(2) $$V_{T(ISFET)}=\;E_{R}+\;\varphi_{LJ}-\;\varphi_{S}+\;X_{EL}+\;\varphi_{F}-\;\frac{E_{F}+\;\frac{E_{G}}{2}}{q}-\;\frac{Q_{S}+\;Q_{D}}{C_{IN}}\;\;\;\;$$

The variables are defined as follows: *E_R_* represents the potential of the reference gate, while *φ_LJ_* denotes the potential at the liquid junction. *φ_S_* corresponds to the potential of the surface charge accumulating on the sensing film. *X_EL_* refers to the surface dipole potential of the electrolyte solution. Regarding the semiconductor properties, *φ_F_* is the Fermi potential, *E_F_* represents the electron affinity of silicon, and *E_G_* signifies the energy band gap. Finally, *Q_S_* denotes the oxide surface charge per unit area, *Q_D_* is the silicon depletion charge per unit area at the threshold voltage, and *C_IN_* stands for the oxide capacitance per unit area. Crucially, an examination of this equation reveals a significant correlation: when DNA molecules induce negative charges on the oxide surface, it invariably leads to an observable increase in the threshold voltage. For the ISFET, the current equation model within the linear region behaves analogously to that of a standard transistor, as illustrated in Equation (3):

(3) $$I_{DS}=\;K_{n}\left[V_{GS}-\;V_{T\left(ISFET\right)}-\;\frac{V_{DS}}{2}\right]V_{DS}\;\;\;$$

Here, *K_n_* represents the transconductance parameter, *V_GS_* is the gate voltage, and V_DS_ denotes the drain-source voltage. Consequently, as the threshold voltage increases, the corresponding drain current decreases.

**Figure 5:**
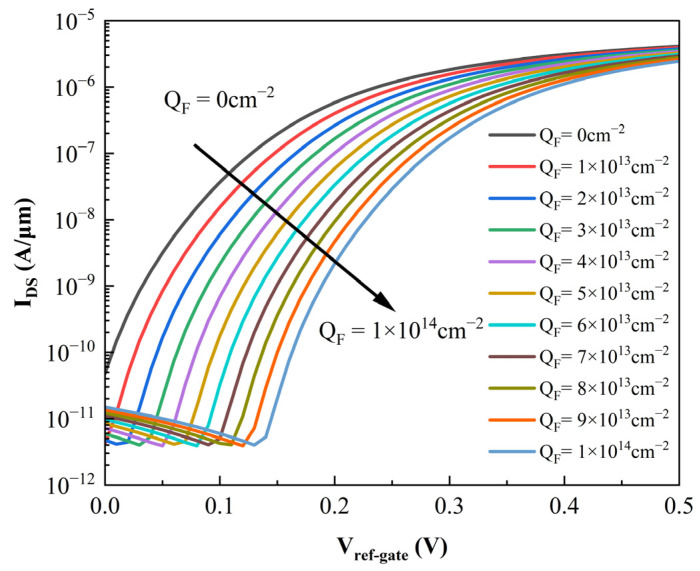
Simulated transfer characteristics (*I_DS_–V_ref-gate_*) of the proposed SU-ISFET biosensor with *V_DS_* fixed at 0.5 V. The plot delineates the shift in current response as the oxide surface negative charge density *Q_F_* increases in magnitude from 0 to 1 × 10^14^ cm^−2^, corresponding to varying concentrations of captured biomolecules.

A complex interplay of variables dictates the probe density on the top oxide layer: the solution ionic strength, interfacial electrostatic potential, the specific nature of the probe—single-stranded DNA (ssDNA) versus double-stranded DNA (dsDNA)—and the immobilization time [25]. Anchored in the experimental benchmarks established by Alexander W. Peter [26], the oxide surface negative charge density *Q_F_* typically spans the magnitude of 10^13^ to 10^14^ cm^−2^. Guided by these findings, we extracted representative values from this range to parameterize our simulations. With the drain-source voltage *V_DS_* fixed at 0.5 V, the resulting transfer characteristics are presented in Figure 5. Here, *V_ref-gate_* denotes the reference gate voltage applied via the electrolyte. A clear trend emerges from the data: as the negative charge density on the top oxide accumulates, the threshold voltage *V_TH_* shifts perceptibly to the right. This behavior aligns perfectly with the theoretical predictions outlined in Equation (2).

**Figure 6:**
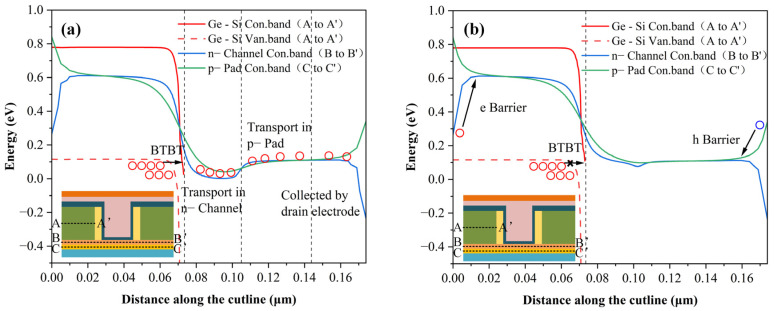
Energy band diagrams spatially resolved across the biosensor structure. (**a**) The baseline equilibrium state in the absence of DNA molecules. (**b**) The modulated state in the presence of DNA, illustrating how the negative charge accumulation alters the band bending profile.

Figure 6 illustrates the internal energy band diagrams of the biosensor under bias conditions of *V_ref-gate_* = 0.15 V, *V_DS_* = 0.5 V. The analysis contrasts the baseline state (absence of DNA molecules) with the detection state, where DNA induces a negative surface charge density of 10^14^ cm^−2^. Analyzing these band profiles confirms that the device operates fundamentally via the BTBT mechanism. The physics of detection unfold as follows: when negatively charged DNA strands hybridize with the probes, they effectively depress the electric potential at the oxide interface. This potential drop propagates, causing a simultaneous reduction in the floating gate voltage. Consequently, the downward band bending within the n+ pocket layer is significantly dampened compared to the DNA-free scenario. This suppression has a direct electrical consequence: the tunneling current in the presence of DNA is markedly lower than in its absence. Remarkably, within a specific *V_ref-gate_* window, the presence of DNA can completely pinch off the channel, forcing the biosensor into a distinct “off” state.

**Figure 7:**
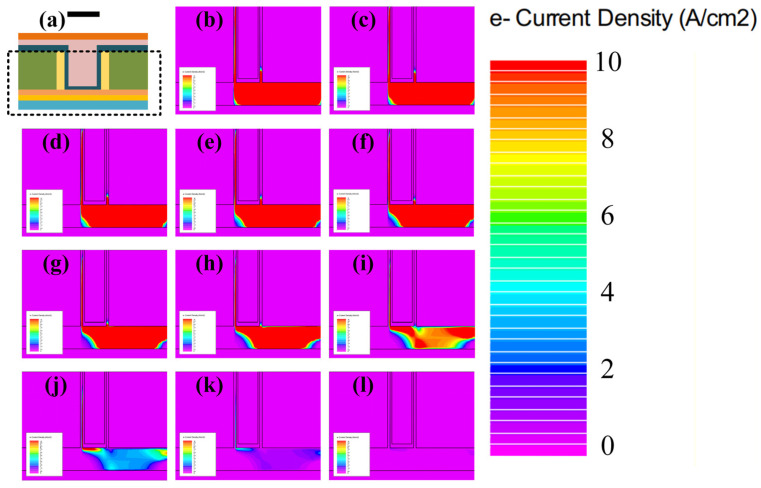
(**a**) Cross-sectional schematic of the SU-ISFET. The region highlighted by the dashed line is magnified in the following figure. Electron current density distribution with surface negative charge of (**b**) 0 cm^−2^ (no DNA detected), (**c**) 1 × 10^13^ cm^−2^, (**d**) 2 × 10^13^ cm^−2^, (**e**) 3 × 10^13^ cm^−2^, (**f**) 4 × 10^13^ cm^−2^, (**g**) 5 × 10^13^ cm^−2^, (**h**) 6 × 10^13^ cm^−2^, (**i**) 7 × 10^13^ cm^−2^, (**j**) 8 × 10^13^ cm^−2^, (**k**) 9 × 10^13^ cm^−2^, (**l**) 1 × 10^14^ cm^−2^.

Figure 7 illustrates the magnitude and spatial distribution of the electron current density within the biosensor under bias conditions of *V_ref-gate_* = 0.15V and *V_DS_* = 0.5 V. The analysis contrasts the DNA-free state with scenarios where DNA molecules induce negative surface charge densities ranging from 10^13^ cm^−2^ to 10^14^ cm^−2^ at the oxide interface. It is observed that the internal current of the SU-ISFET decreases monotonically as the surface charge density increases. Specifically, at a charge density *Q_F_* of 10^14^ cm^−2^, the conductive tunneling junction within the biosensor is effectively depleted. This observation is supported by the energy band diagrams in Figure 6, which indicate that the BTBT process at the Ge-Si heterojunction is inhibited under this condition *Q_F_* = 10^14^ cm^−2^. Consequently, the residual current in this state is primarily composed of thermally excited electrons originating from the channel and Pad layers. Furthermore, the band diagrams in Figure 6 reveal that the doping concentration discrepancy between the Pad and channel layers induces specific potential barriers for both electrons and holes. These barriers effectively suppress the leakage current arising from thermal excitation. Conversely, in the absence of DNA molecules, Figure 7 demonstrates that the electron current is dominated by the BTBT current generated at the Ge-Si interface. During operation, valence band electrons tunnel across the junction into the heavily doped n-type pocket layer. Subsequently, driven by the drain bias, these carriers traverse the channel and Pad layers towards the drain region, where they are ultimately collected.

### 3.2. Sensitivity Analysis for DNA Detection

The characterization of biosensor performance is fundamentally anchored in tracking the magnitude of shifts in key electrical parameters [27]. To quantify this sensitivity, we adopt a generalized expression formulated in the following equation:

(4) $$S_{A}\left(\%\right)=\left|\frac{A^{bio}-A^{air}}{A^{air}}\right|\times 100$$

Here, *A* represents a generalized variable denoting any electrical parameter of the device. The sensing performance of this architecture is largely attributed to the ultra-low Subthreshold Swing (SS) of the TFET. This characteristic ensures that, at a fixed reference gate voltage (*V_ref-gate_*), a distinct variation in drain current (*I_DS_*) is observed between the presence and absence of DNA molecules. Consequently, *I_DS_* is adopted as a metric to quantify the current sensitivity (*S_IDS_*), which is defined as follows:

(5) $$S_{IDS}\left(\%\right)=\left|\frac{{I_{DS}}^{bio}-{I_{DS}}^{air}}{{I_{DS}}^{air}}\right|\times 100$$

**Figure 8:**
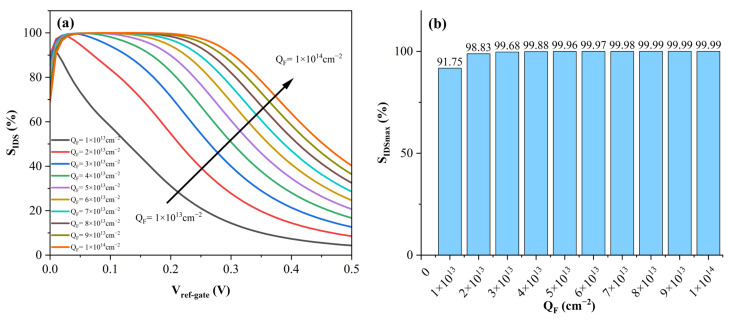
(**a**) Current sensitivity characteristics of the biosensor with respect to reference gate voltage *V_Ref-Gate_*. The plot displays the evolution of *S_IDS_* under different oxide surface charge densities *Q_F_*, demonstrating the correlation between charge magnitude and the effective sensitivity range. (**b**) Dependency of maximum current sensitivity *S_IDSmax_* on the oxide surface charge density. The histogram presents the peak sensitivity values achievable across a range of *Q_F_* from 10^13^ to 10^14^ cm^−2^, illustrating a distinct saturation trend towards 100% as the charge concentration rises.

Within this mathematical formulation, *I_DS_^bio^* and *I_DS_^air^* denote the source-drain currents measured under identical *V_ref-gate_* conditions, corresponding to the electrolyte containing target DNA molecules and the DNA-free baseline, respectively. Figure 8a profiles the evolution of the biosensor’s current sensitivity (*S_IDS_*) as a function of the reference gate voltage (*V_ref-gate_*), sweeping the oxide surface charge density (*Q_F_*) from 10^13^ to 10^14^ cm^−2^. Analysis of the low-voltage regime (*V_ref-gate_* < 0.05 V) reveals a sharp dynamic: the sensitivity surges rapidly, peaking at approximately 100%, before undergoing a gradual decay as the gate voltage increases. Specifically, under a high surface negative charge density of -10^14^ cm^−2^, the sensitivity exhibits remarkable retention—maintaining a value as high as 99.99% at *V_ref-gate_* = 0.2 V—and sustains a robust level of ≈ 90% even as the voltage extends to 0.3 V. Crucially, the surface charge density exerts a profound influence on the retention characteristics of the sensitivity profile. As *Q_F_* intensifies from 10^13^ to 10^14^ cm^−2^, the sensitivity curves display a distinct broadening effect. At lower charge concentrations, *S_IDS_* suffers a precipitous drop at higher gate voltages; however, elevated levels of *Q_F_* effectively mitigate this decay, preserving high sensitivity over a wider voltage window. Exploiting this characteristic, the device can be tuned for the precise quantification of varying DNA concentrations. Parallel to this, *Q_F_* dictates the maximum achievable sensitivity (*S_IDmax_*), as delineated in Figure 8b. The peak value initiates at 91.75% for a *Q_F_* of 10^13^ cm^−2^ and climbs to near-saturation at 99.99% once the negative charge density reaches 8 × 10^13^ cm^−2^. Fundamentally, the magnitude of this surface charge is intrinsically linked to both the density of the immobilized probes and the concentration of the DNA solution. Consequently, the optimization of surface probe functionalization and the precise formulation of the DNA analyte serve as critical determinants of the biosensor’s ultimate sensitivity.

Drawing upon the analysis in Figure 6, it is evident that the electrostatic charge—arising from the hybridization of target DNA with immobilized probes—modulates the potential at the oxide interface, thereby exerting a substantial impact on the threshold voltage (*V_TH_*). Reflecting this physical coupling, the shift in *V_TH_* is widely established in the literature as a critical figure of merit for benchmarking biosensor sensitivity [28]. This metric is quantitatively defined by the following equation:

(6) $$S_{VTH}(\%)=\left|\frac{{V_{TH}}^{bio}-{V_{TH}}^{air}}{{V_{TH}}^{air}}\right|\times 100$$

In this expression, *V_TH_^bio^* and *V_TH_^air^* denote the threshold voltages of the biosensor measured in the presence of target biomolecules and in the DNA-free baseline state, respectively.

**Figure 9:**
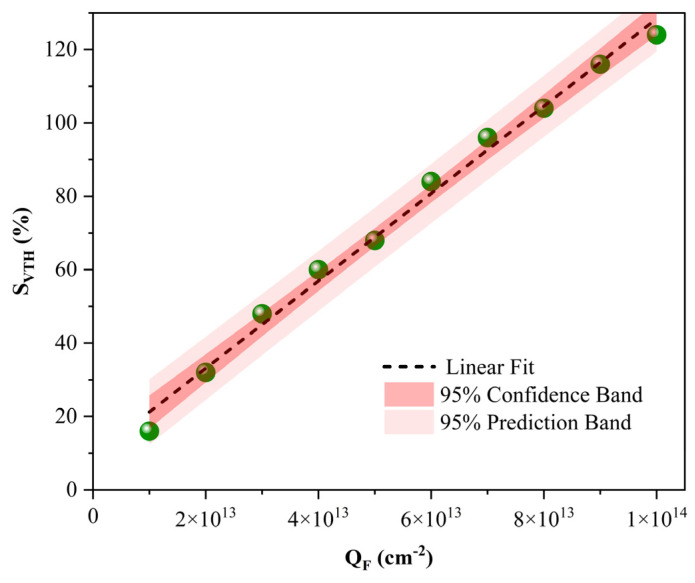
Linear dependency of the threshold voltage sensitivity (*S_VTH_*) on the oxide surface charge density (*Q_F_*). The plot illustrates a strict linear correlation, where the sensitivity scales proportionally with the magnitude of the negative surface charge.

Figure 9 presents the variation profile of the threshold voltage sensitivity (*S_VTH_*) as a function of the oxide surface charge density. In distinct contrast to the behavior observed in current sensitivity, *S_VTH_* exhibits a remarkably linear upward trend. This linearity is attributed to the progressive elevation of the threshold voltage induced by the accumulation of negative surface charge—a phenomenon that aligns precisely with the theoretical predictions of Equation (2). Quantitatively, as the negative charge concentration scales from 10^13^ to 10^14^ cm^−2^, the sensitivity (*S_VTH_*) escalates dramatically, surging from 16% to 124%. This also indicates that a high oxide surface charge density induces a significant shift in the threshold voltage of the SU-ISFET, thereby facilitating the detection of DNA molecules.

To benchmark the proposed device against other recently reported biosensors, the off-state current sensitivity (*S_IOFF_*) was also evaluated using the same methodology defined previously. Table 2 presents a comparative analysis of sensitivity metrics from recent literature [29,30,31,32]. The results indicate that the SU-ISFET exhibits superior performance characteristics, positioning it as one of the top-performing biosensors among those compared.

### 3.3. Impact of Geometric Parameters on Sensing Performance

**Figure 10:**
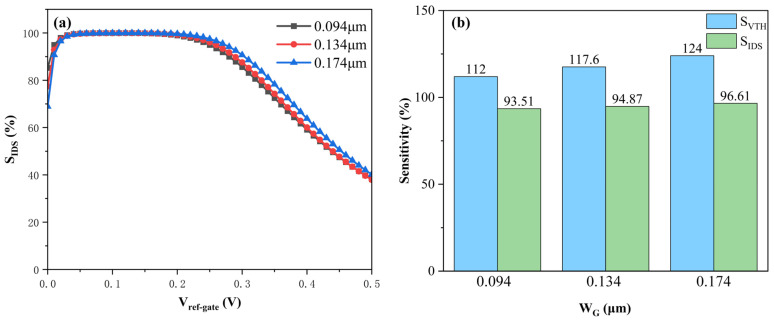
Sensitivity modulation via geometrical scaling of the top gate oxide (W_G_). (**a**) Evolution of the *S_IDS_* curves under different W_G_ dimensions, highlighting the enhanced retention of sensitivity at higher voltages for longer oxide lengths. (**b**) Extracted values for *S_VTH_* and *S_IDS_*.

We proceeded to investigate the critical role of the top gate Al_2_O_3_ oxide width (W_G_) in dictating the biosensor’s performance metrics. To isolate the impact of this specific parameter during simulation, W_G_ was varied as the independent variable, while the total device width (W_BOX_) remained constant. The simulation boundary conditions assumed a detection state characterized by a surface charge density of 10^14^ cm^−2^ and a drain bias *V_DS_* of 0.5 V. The resulting performance evolution is visualized in Figure 10. A salient observation from the data is the broadening of the current sensitivity profile as the top gate oxide width expands. Notably, the rate of sensitivity decay is significantly retarded with increasing W_G_. Complementing this analysis, Figure 10b delineates the dependency of the threshold voltage sensitivity (*S_VTH_*) on the oxide width, alongside the current sensitivity (*S_IDS_*) extracted at a specific operating point of *V_ref-gate_* = 0.26 V. The data reveals a clear monotonic enhancement: both *S_IDS_* and *S_VTH_* amplify as the oxide layer elongates. When W_G_ increases to its maximum limit (W_BOX_), both the current sensitivity and threshold voltage sensitivity attain their maximum values. This performance enhancement is primarily attributed to the geometric expansion of the effective sensing area. A wider W_G_ translates to a larger surface footprint, thereby accommodating a greater aggregate of negative charges upon DNA hybridization. The extended top gate geometry also fortifies the capacitive coupling between the reference gate and the channel. This superior electrostatic control effectively reduces the SS. A reduced SS implies a sharper transition between off-state and on-state currents, which—in synergy with the increased charge capture—significantly amplifies the biosensor’s overall sensitivity.

### 3.4. Impact of Gate Dielectric Materials

As the SU-ISFET biosensor operates fundamentally via the BTBT mechanism, the dielectric constant of the gate oxide material plays a pivotal role in determining the device’s intrinsic electrical characteristics. Specifically, it exerts a significant impact on key figures of merit, including the SS and the on/off current ratio. For TFETs, the carrier tunneling probability is quantitatively governed by the WKB approximation [33]:

(7) TWKB∝exp(−42m∗Eg323eħ(Eg+ΔΦ)εcεoxtoxtc)ΔΦ

In this expression, m* represents the effective carrier mass, *E_g_* denotes the bandgap, and *ħ* is the reduced Planck constant. ΔΦ signifies the energy difference across the tunneling junction, while *ε_c_* and *t_c_* correspond to the dielectric constant and thickness of the channel, respectively. *t_ox_* and *ε_ox_* refer to the thickness and dielectric constant of the gate oxide. According to this analytical relationship, a higher gate oxide dielectric constant (*ε_ox_*) leads to a substantial increase in the tunneling probability. Consequently, employing a high-k material such as HfO_2_ as the gate dielectric not only facilitates a larger tunneling current but also yields a steeper SS. Table 3 summarizes the performance metrics—specifically, the maximum current sensitivity (*S_IDSmax_*) and threshold voltage sensitivity (*S_VTH_*)—for biosensors utilizing SiO_2_, Al_2_O_3_, and HfO_2_ as gate dielectrics. The data clearly demonstrates a positive correlation: as the dielectric constant of the gate material increases, both *S_IDSmax_* and *S_VTH_* exhibit progressively superior values.

### 3.5. Noise Analysis

In complex and practical bio-sensing applications, biosensors are inevitably subject to noise interference originating from both the solution environment and peripheral circuits. Such background noise poses a significant challenge for high-sensitivity biosensors. While striving for high sensitivity to amplify weak biological signals, these devices often inadvertently introduce pronounced nonlinear distortion effects, thereby compromising measurement accuracy [34]. Theoretically, to achieve a wide linear dynamic range, the transfer characteristic curve of the device must remain highly linear. In this section, the third-order transconductance (*g_m_*_3_) is introduced as a metric for evaluating linearity. A smaller magnitude of *g_m_*_3_ indicates fewer high-order harmonic components, corresponding to superior noise immunity and signal linearity [35].

**Figure 11:**
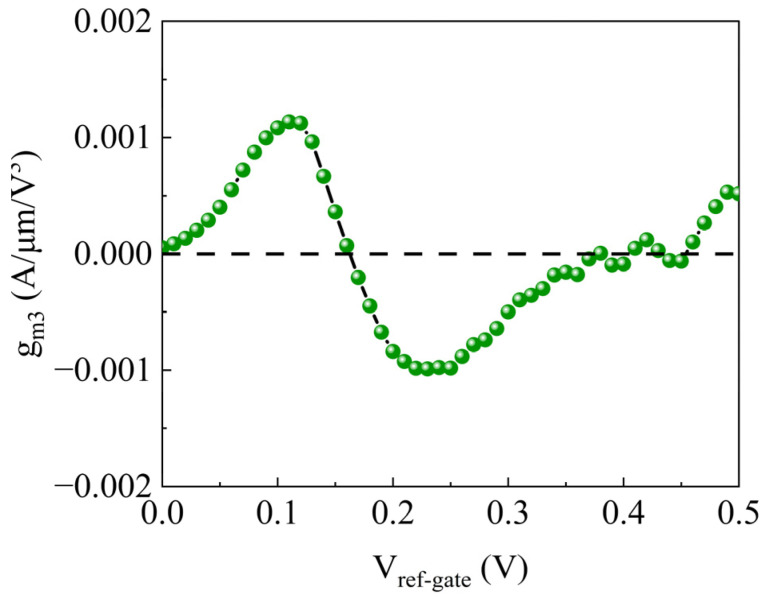
The linearity coefficient (*g_m_*_3_) of the SU-ISFET versus the reference gate voltage. The dashed line represents the zero reference line.

As illustrated in Figure 11, within the studied reference gate voltage range, the linearity coefficient curve of the ion-sensitive symmetric U-shaped TFET biosensor exhibits two primary peaks (upper and lower), showing significant non-monotonic oscillatory behavior and distinct zero-crossing points. Notably, the upper peak is the most prominent, reaching a maximum amplitude of 0.0012 A/μm/V^3^. These zero-crossing points are critical for RF applications; at these specific bias voltages, *g_m_*_3_ theoretically vanishes, thereby eliminating the dominant third-order nonlinear components. Consequently, the proposed biosensor demonstrates excellent noise tolerance and interference immunity in complex biochemical environments. This capability to maintain high linearity during the sensing process effectively ensures the signal-to-noise ratio (SNR) and accuracy of the detected signals.

### 3.6. Process Reliability

For the SU-ISFET, the core band-to-band tunneling (BTBT) structure consists of a p-type Ge source region and an n-type Si pocket layer. Due to the lattice mismatch between Ge and Si, a certain density of interface states is inevitably induced at the Si/Ge heterojunction interface. These states typically act as electrically active centers, serving either as carrier recombination centers or as traps that capture charges to form interface trapped charges. Theoretically, such states can potentially perturb the energy band bending and electric field distribution at the heterojunction.

**Figure 12:**
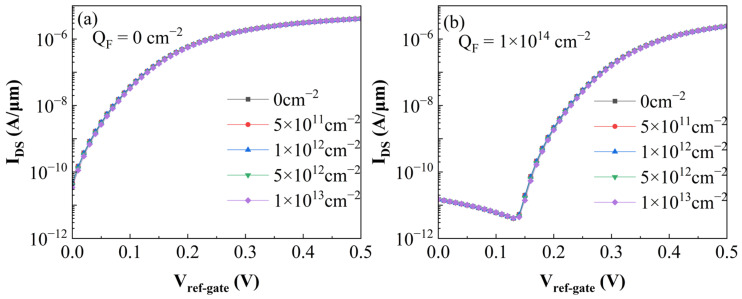
Transfer characteristic curves of the biosensor with different electron trap densities at the heterojunction. (**a**) Without DNA molecules in the solution. (**b**) With DNA molecules bound to the oxide surface, inducing a charge density of 10^14^ cm^−2^.

**Figure 13:**
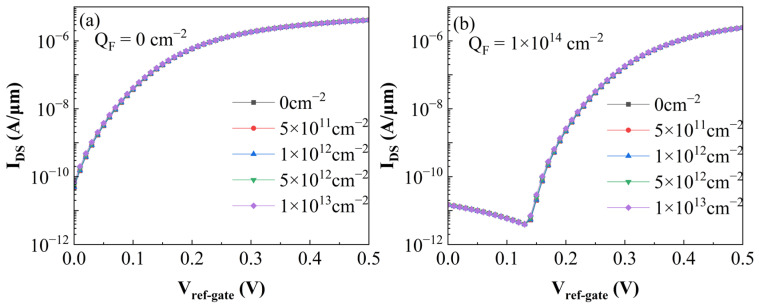
Transfer characteristic curves of the biosensor with varying hole trap densities at the heterojunction. (**a**) In the absence of DNA molecules. (**b**) With DNA molecules immobilized on the Al_2_O_3_ surface, inducing a charge density of 10^14^ cm^−2^.

To evaluate the impact of the aforementioned non-ideal factors on the biosensor, this study introduced trap charges of varying types and areal densities at the Si/Ge heterojunction interface. Figure 12 and Figure 13 clearly illustrate the variations in the biosensor’s transfer characteristic curves under the presence of electron and hole traps, respectively. Observing the simulation results, it is evident that the transfer characteristics of the device exhibit negligible variations. This phenomenon is attributed to the fact that both the p-type Ge source and the n-type Si pocket layer on either side of the tunneling junction are heavily doped, resulting in an extremely narrow depletion region and a high carrier concentration. Consequently, the density of trap charges formed by interface state capture is significantly lower than the ionized impurity charge density. Therefore, the energy band bending and tunneling probability at the junction are predominantly governed by the doping profile. The additional electric field induced by interface traps is effectively screened by the high concentration of free carriers, thereby exerting minimal influence on the biosensor’s performance.

## 4. Conclusions

In this paper, a novel SU-ISFET biosensor is proposed and systematically investigated using Silvaco Atlas TCAD simulation. The investigation reveals that the steep subthreshold characteristics facilitated by the BTBT mechanism significantly enhance the biosensor’s detection capability for DNA molecules. Simulation results demonstrate outstanding sensitivity metrics, specifically achieving a maximum current sensitivity *S_IDSmax_* of 99.99% and a threshold voltage sensitivity *S_VTH_* of 124%. Furthermore, the paper analyzed the impact of specific design parameters, including the top gate oxide length (W_G_) and gate dielectric materials, providing clear guidelines for performance optimization. In addition, the noise characteristics and process reliability were strictly evaluated to ensure the device’s stable operation in practical scenarios. Consequently, the SU-ISFET is validated as a promising candidate for future low-power biomedical detection systems.

## Figures and Tables

**Table 1:** Device parameters of the SU-ISFET biosensor.

Parameter	Symbol	Value	Units
Height of Source/Drain	H_S_/H_D_	40	nm
Width of Gate	W_G_	174	nm
Height of the Channel	H_C_	5	nm
Height of the Pad	H_Pad_	5	nm
Height of the SiO_2_ Box	H_Box_	5	nm
Width of the Box	W_Box_	174	nm
Gate oxide layer thickness	T_OX_	2	nm
Height of Al_2_O_3_ oxide layer	H_OX2_	2	nm
p+ Source/Drain doping concentration	*N_S_*/*N_D_*	2.5 × 10^19^	cm^−3^
n+ Pocket doping concentration	*N_P_*	3.1 × 10^19^	cm^−3^
n− Channel doping concentration	*N_C_*	1 × 10^15^	cm^−3^
p− Pad doping concentration	*N_Pad_*	1 × 10^15^	cm^−3^

**Table 2:** Comparison of Sensitivity among various biosensor architectures.

Refs./Method	Architecture	*S_IOFF_* (%)	*S_VTH_* (%)
[29]/Simulation	DG junction-less TFET	99.99	28.57
[30]/Simulation	FinFET	98.4	26.34
[31]/Simulation	InGaAs/Si HTFET	90	-
[32]/Simulation	Vertical dual DL TFET	98.58	50.27
This Work	SU-ISFET	99.99	124

**Table 3:** Summary of sensitivity metrics for biosensors utilizing different gate dielectric materials.

Materials	*S_IDSmax_* (%)	*S_VTH_* (%)
SiO_2_	97.54	23.08
Al_2_O_3_	99.38	57.14
HfO_2_	99.99	124

## Data Availability

The original contributions presented in this study are included in the article. Further inquiries can be directed to the corresponding author.

## Abbreviations

The following abbreviations are used in this manuscript:

ISFET	Ion-Sensitive Field-Effect Transistor
TFET	Tunnel Field-Effect Transistor
BTBT	Band-to-Band Tunneling
SS	Subthreshold Swing
MOSFET	Metal-Oxide-Semiconductor Field-Effect Transistor
CMOS	Complementary Metal-Oxide-Semiconductor
FBFET	Feedback Field-Effect Transistor
SU-ISFET	Symmetric U-shaped gate tunnel FET-ISFET
BSA	Bovine Serum Albumin
DNA	Deoxyribonucleic acid
CVD	Chemical Vapor Deposition
CMP	Chemical Mechanical Polishing
ALD	Atomic Layer Deposition
TAT	Trap-Assisted Tunneling
BGN	Bandgap Narrowing
CVT	Lombardi Mobility Model
TCAD	Technology Computer Aided Design
WKB	Wentzel–Kramers–Brillouin approximation
